# Revival of Brock's Operation for Intermediary Palliation of Fallot's Tetralogy in Children Anatomically Unsuitable for One-Stage Total Correction of the Anomaly: Interim Results of Two Cases

**DOI:** 10.7759/cureus.39255

**Published:** 2023-05-20

**Authors:** Vishal V Bhende, Tanishq S Sharma, Krishnan Ganapathy Subramaniam, Deepakkumar V Mehta, Jigar P Thacker, Ashwin S Sharma, Amit Kumar, Gurpreet Panesar, Kunal A Soni, Kartik B Dhami, Nirja Patel, Hardil P Majmudar, Sohilkhan R Pathan

**Affiliations:** 1 Pediatric Cardiac Surgery, Bhanubhai and Madhuben Patel Cardiac Centre, Bhaikaka University, Gokal Nagar, Karamsad, IND; 2 Community Medicine, SAL Institute of Medical Sciences, Ahmedabad, IND; 3 Pediatric Cardiac Surgery, Sri Padmavati Pediatric Heart Centre, Sri Venkateswara Institute of Medical Sciences (SVIMS) Campus, Tirupati, IND; 4 Radiodiagnosis & Imaging, Pramukhswami Medical College & Shree Krishna Hospital, Bhaikaka University, Gokal Nagar, Karamsad, IND; 5 Pediatrics, Pramukhswami Medical College & Shree Krishna Hospital, Bhaikaka University, Gokal Nagar, Karamsad, IND; 6 Internal Medicine, Gujarat Cancer Society Medical College, Hospital and Research Centre, Ahmedabad, IND; 7 Pediatric Cardiac Intensive Care, Bhanubhai and Madhuben Patel Cardiac Centre, Bhaikaka University, Gokal Nagar, Karamsad, IND; 8 Cardiac Anaesthesiology, Bhanubhai and Madhuben Patel Cardiac Centre, Bhaikaka University, Gokal Nagar, Karamsad, IND; 9 Clinical Research Services (CRS), Bhanubhai and Madhuben Patel Cardiac Centre, Bhaikaka University, Gokal Nagar, Karamsad, IND

**Keywords:** brock's procedure, pulmonary stenosis, closed pulmonary infundibular resection, pulmonary valvar incompetence, palliation, tetralogy of fallot

## Abstract

One-stage total correction is known to be anatomically unsuitable for correcting tetralogy of fallot (TOF) in a certain proportion of children. Surgeons are thus faced with dilemmas regarding which preliminary operation for the anomaly to do first. Brock's primary postulation suggests that pulmonary trunk and annulus enlargement leading to the correction of the outflow obstruction will favor the subsequent total correction. In line with this, the current article presents two patients who were 6 months and 5 years old. The first patient underwent primary Brock's operation while the second patient had a blocked modified Blalock-Taussig's shunt (MBTS) done off-pump. Following the discontinuation of anti-platelet medications, the MBTS blocked and the patient was subsequently considered for secondary Brock’s operation. The outcome of both procedures involved the patients’ discharge with uneventful hospital stays and regular follow-ups at specified intervals. Thus, Brock's operation is an excellent preliminary palliative procedure for one-stage total correction of TOF. There is a need to revive 'Brock's procedure' for patients with TOF and poor pulmonary artery anatomy as the procedure of choice. The first direct intra-cardiac operation aimed at directly addressing the pathological anatomy on its Diamond Jubilee Year.

## Introduction

The choice of surgical procedures for tetralogy of Fallot (TOF) is often hindered by the right ventricular outflow tract (RVOT) obstruction in children having marked pulmonary trunk and annular hypoplasia. This presents a major concern for surgeons in the management of children with TOF. Blalock-Thomas-Taussig (BTT) shunt was the first pioneering extra-cardiac procedure aimed at palliating blue babies. The shunt emanated from an unsuccessful attempt at creating an animal model of pulmonary hypertension by anastomosing the subclavian artery to the pulmonary artery [1] .

Following the introduction of the BTT shunt in 1944, it caught the attention of surgeons as well as lay people and became an immensely popular method of palliation until it was overshadowed by direct corrective surgery. Around the same time in 1947, Brock made a series of clinical observations as well as external and internal surgical procedures on patients with pulmonary stenosis and TOF. It was discovered that following left pneumonectomy, a cardioscope can be passed through the left pulmonary artery to inspect the pulmonary valve directly. The cardioscope used in the management of blue babies in 1948 was found to function better when inserted through the ventricle because of its funnel shape and the pinhole nature of the pathology [2].

Although the procedure did not gain similar popularity as the BTT shunt procedure, it remained the first direct intra-cardiac procedure before the advent of cardiopulmonary bypass and echocardiography. Notably, it was considered and attempted due to a series of clinical and autopsy studies and not based on experimental animal studies. The present case studies was meant to showcase and verify the effects of an age-long Brock’s procedure as palliative preliminary care for children with TOF associated with pulmonary stenosis.

## Case presentation

The Research and Ethics Committee (IEC-2) of the HM Patel Centre for Medical Care and Education, Anand, Gujarat, India granted approval for this case study (Ref. No. IEC/BU/2022/Cr.21/99/2022 dated 28.06.2022). Written informed consent was granted by the parents of the participating children before undergoing the surgical procedure.

Case 1

**Figure 1:**
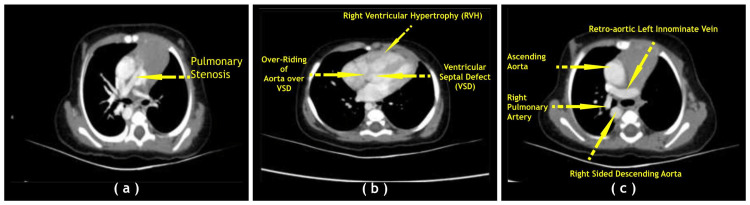
(a) CT axial image showing pulmonary stenosis and hypoplastic pulmonary arteries in a 6-month-old baby, who underwent primary Brock’s operation; (b) Axial image showing ventricular septal defect with over-riding the aorta as well as right ventricular enlargement; (c) Axial image showing retro-aortic left innominate vein and right-sided descending aorta. Image credits: Dr. Deepakkumar V. Mehta

The first case is a 6-month-old female child, weighing 5.8 kg, referred to the Cardiac Centre for further management of TOF and pulmonary stenosis. The child was delivered at term through a normal vaginal delivery weighing 2.75 kg and cried immediately after birth. The baby has been maintained with a saturation of 65% on room air. The girl was evaluated with 2-D echocardiography and cardiac CT dynamic study. The 2-D echocardiography revealed TOF with a large sub-aortic malaligned ventricular septal defect, bidirectional shunt, severe sub-valvar, valvar, and supra-valvar pulmonary stenosis. No pulmonary valve regurgitation was observed. The branch pulmonary arteries were small [right pulmonary artery, RPA: 6 mm; proximally (z score + 0.12); left pulmonary artery, LPA: 4.1 mm; proximally (z score - 1.57); main pulmonary artery, MPA: 6.5 mm (z score - 2.6); pulmonary annulus: 4.6 mm (z score -5.6)]. A right aortic arch with mirror-image branching and a retro-aortic innominate vein were also observed. The patient also underwent a cardiac dynamic CT scan study which profiled the RPA and LPA as 6 and 4.1 mm, respectively. A few small 2-3-mm mediastinal aortopulmonary collaterals were noted from the descending thoracic aorta at a 3° clock position (Figure 1). 

Case 2

This case is a 5-year-old child weighing 15 kg, who underwent a secondary Brock's operation. The child was delivered at term through a normal vaginal delivery and weighed 3 kg. A modified Blalock-Taussig's shunt, measuring 4 mm. was performed off-pump at 1.5 years of age for TOF with pulmonary stenosis, and major aortopulmonary collateral arteries (MAPCAs). Moreover, the patient had an uneventful primary surgery and maintained room air saturations of 72%-75%. In the early part of 2022, the child's parents discontinued the postoperative antiplatelet medication acetylsalicylic acid (Ecosprin), 75 mg, following the primary surgery. The child started developing gradual cyanosis, resulting in shunt blockage, due to the abrupt discontinuation of antiplatelet medications.

**Figure 2:**
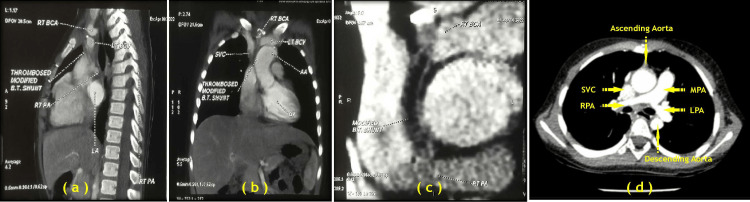
(a) CT sagittal reformation of blocked 4-mm sized PTFE-modified Blalock-Taussig shunt performed in a 5-year-old female child with tetralogy of Fallot with pulmonary stenosis with MAPCAs in October 2018; (b) CT coronal reformation of blocked modified Blalock-Taussig shunt; (c) CT curved oblique reformation of blocked modified Blalock-Taussig shunt; (d) Contrast CT axial image at the level of pulmonary arteries. BCA, Brachio-cephalic artery; BCV, Brachio-cephalic vein; AA, ascending aorta; SVC, superior vena cava; LA, left atrium; LV, left ventricle; PTFE, polytetrafluoroethylene; MAPCAs, major aorto-pulmonary collateral arteries; MPA, main pulmonary artery; RPA, right pulmonary artery; LPA, left pulmonary artery Image Credits: Dr. Deepakkumar V. Mehta

The child was re-evaluated at the Cardiac Centre Clinic using 2-D echocardiography and cardiac CT dynamic study which confirmed the following. A long segmental thrombosis was found in the modified Blalock-Taussig's shunt (MBTS) having luminal patency only at its superior and inferior ends. An adequate size of the pulmonary trunk, probably due to post-stenotic dilatation, was observed with a small diameter of the proximal left and distal right pulmonary arteries [RPA: proximal 9.2 mm, distal 8.3 mm (z score - 0.73), LPA: proximal 7.5 mm, distal 9.6 mm (z score - 0.51), MPA: proximal 15.5 mm, distal 15.1 mm (z score +0.04), pulmonary valve/annulus: 7.6 mm (z score - 4.46)] (Figure 2).

As per the SARS-CoV2 (COVID-19) protocol in place, the patient underwent a polymerase chain reaction for SARS-CoV2 and was found to be negative. The characteristics of both cases are shown in Table 1.

**Table 1:** Characteristics of the cases at the time of surgery. CPB, cardiopulmonary bypass; ACC, aortic cross-clamp; ICU, intensive care unit; RVOT, right ventricular outflow tract; LPA, left pulmonary artery; PTFE, polytetrafluoroethylene; pRV/pLV, peak systolic pressure ratio of right ventricle and left ventricle

Parameters	Case 1	Case 2
Age	6 Months	5 years
Sex	Female	Female
Weight	5.8 kg	15 kg
Surgery	Primary Brock’s operation	Primary modified Blalock-Taussig’s shunt using PTFE 4 mm graft done in October 2018; secondary Brock’s operation
Ventilator hours	50 h	25 h
Length of ICU stay	6 days	5 days
Hospital stay	12 days	10 days
CPB time	156 min	133 min
ACC time	86 min	75 min
pRV:pLV pressure	0.8	0.8
2-D echocardiography at discharge	Small patch in RVOT (the gradient across pulmonary valve is 56 mmHg. Good flow in the right pulmonary artery (a trickle of flow was observed in the left pulmonary artery)	Good flow from right ventricle to the branch pulmonary arteries peak gradient across the right ventricular outflow tract is 50 mmHg. Moderate to severe pulmonary stenosis; mild pulmonary valve regurgitation

Technique of Brock's operation

**Figure 3:**
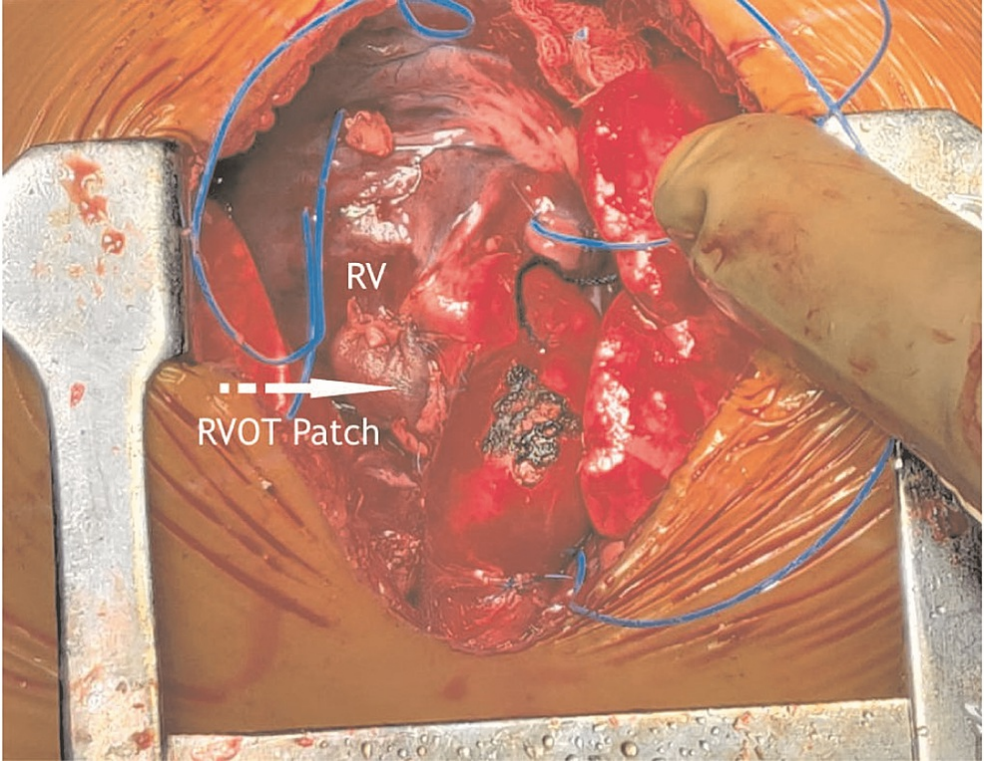
RVOT patch in Case 1 (primary Brock’s operation) at 6 months old. RVOT, right ventricular outflow tract; RV, right ventricle Image Credits: Dr. Vishal V. Bhende

**Figure 4:**
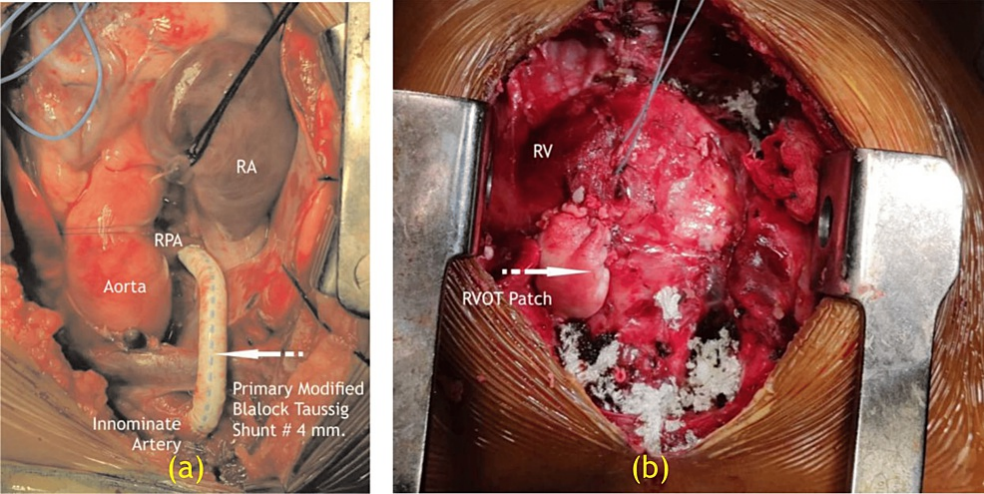
(a) Primary modified Blalock-Taussig shunt #4 mm done in case 2 at 1.5 years old. (b) RVOT patch in case 2 (secondary Brock’s operation). RA, right atrium; RV, right ventricle; RPA, right pulmonary artery Image Credits: Dr. Vishal V. Bhende

Brock [2], in 1948, reported a surgical procedure for correcting congenital pulmonary stenosis. Even if shunting procedures had their advantages, surgically and physiologically attacking the stenotic valve or the infundibular obstruction directly would be far better than to merely perform a bypass procedure. Doyen [3] had attempted direct vision of what was believed to be a stenosed pulmonary valve in 1913. Thereafter, no further attempts were done until the development of the Brock's operation. Originally, Doyen tried to approach the valve via the LPA, first observing the anatomic situation via a cardioscope. However, this proved to be unsatisfactory from the point of view of the patient and surgeon. Therefore, the remaining avenue through the right ventricle was employed just as Doyen had previously used it years earlier. Figures 3-4 are intra-operative images of case 1 and case 2, respectively. The surgical summary of Brock's operation has been shown in Video 1 and Table 2.

**Video 1:** Brock's operation 3D medical animation. Video Credits: Dr. Vishal V. Bhende

 

**Table 2:** Surgical summary of Brock's operation. RVOT, right ventricular outflow tract; RV, right ventricle; PTFE, polytetrafluoroethylene; CPB, cardiopulmonary bypass

S. No.	Steps of Brock’s operation
01	Brock's operation can be performed on cardiopulmonary bypass with aorta, right atrial single cannula or bicaval cannulation, cardioplegia can be given at the discretion of operating surgeon.
02	The RVOT should be sized just to the required size of the Hegar dilator to prevent pulmonary flooding and RV distension due to excessive pulmonary regurgitation.
03	The RVOT patch should be taut and PTFE patch/bovine pericardium can be used to retain the native pericardium to help in subsequent corrective surgery.
04	A non-distensible RVOT patch also helps in ensuring that the energy of RV contraction is transmitted to the distal pulmonary vascular bed rather than expended in distending a redundant RVOT patch.
05	RV pressure should be just sub-systemic and there should be pulsatile pulmonary artery flow.
06	When performing on CPB with beating heart, to improve safety the root can be kept on continuous suction with head down to prevent air embolism.
07	Cardioplegia can be used at the discretion of the operating surgeon.

Both cases had an uneventful postoperative recovery (Table 1). Moreover, neither occurrence of mortality nor complications was observed. At the time of discharge and annual follow-ups, cases 1 and 2 maintained room air saturations of 80%-85% and 82%-88%, respectively. Both patients maintained a peak pressure gradient across the RVOT of 56 (case 1) and 50 (case 2) mmHg. This observation was consistent at discharge and thereafter at 6 and 3-month follow-up intervals for cases 1 and 2, respectively.

Both patients had mild pulmonary valve regurgitation on 2-D echocardiography which was evaluated at 3 months, 6 months, and annual visits. No heart blocks were noted and both patients maintained normal sinus rhythm on electrocardiography (ECG) at discharge.

## Discussion

Total repair using a single-stage process remains the greatest surgical procedure for TOF if it can be done. However, concerns were noted about the choice of preliminary palliative operations followed by subsequent total repair. Correction of RVOT obstruction by Brock's procedure is in accordance with a true partial correction which is usually associated with a marked clinical improvement of the affected child. Brock's surgical operation was initially performed in 1948 [2] in order to address a deadly challenge of pulmonic stenosis -- one of the conditions in TOF. Closed pulmonary valvotomy was another procedure separately elucidated by Sellors in the same year [4]. The first three cases described by Brock were three children who had TOF and their procedures were undertaken via the LPA, with poor outcomes [5].The Brock’s technique was subsequently refined by reaching the pulmonary valve via a closed right ventriculotomy. However, because the background infundibular stenosis was not addressed, Brock created a punch method to resect the stenosed infundibulum, leading to better outcomes among five children [5]. In 1955, he published a case study involving 150 children having TOF where the punch approach was carried out whether combined or not with pulmonary valvotomy [6]. Gerlis et al. [6] reported that a patient who had Brock’s technique at 4 years of age lived for 43 years, but ultimately expired at 47 years old due to worsening biventricular failure and pulmonary hypertension. Anastomotic procedures, e.g. Blalock-Taussig, Pott's, and Waterston shunts, although provide satisfactory symptomatic relief, are merely temporary measures with regard to improving survival; and they result in the creation of an additional anatomical anomaly combined with those already existing. Anastomotic procedures also carry a mortality rate that varies from 36% (Taussig, 1969) to 29% (Cole, Muster, Fixler, and Paul, 1971) and 21% (Bernhard, Jones, Friedberg, and Litwin, 1971). There was no mortality in our cases. Hitherto, the oldest reported individual who survived after undergoing Brock's technique was reported by Manchikalapudi and Klugherz [7]. This subject had the technique at 28 years old and survived for 51 more years following the initial procedure. The advantages and disadvantages of Brock's operation are shown in Table 3.

**Table 3:** Advantages and disadvantages of Brock's operation [8]. PAs, pulmonary arteries; RV, right ventricle; BTT, Blalock-Thomas-Taussig; CPB, cardio-pulmonary bypass; VSD, ventricular septal defect

Advantages	
Provides antegrade flow through the natural route which can help in uniform growth of branch PAs without distortion	
Infundibular and RV cavities can also grow which makes the subsequent surgery safer	
Unlike the BTT shunt, which develops acquired infundibular obstruction, distortion of branch PAs and possibly shunt obstruction over time; results of Brock’s procedure provide much durable palliation which improves with time	
The procedure needs not be taken down at the time of corrective operation	
Can be performed with limited pericardiotomy and it prepares the pulmonary bed gradually for increased pulmonary blood flow making the subsequent corrective surgery safer	
Possibly less coronary steal exists due to decreased diastolic runoff	
Can be performed in a limited time without cardioplegia and the need to dissect or clamp the branch pulmonary arteries, reduces the risk of low cardiac output, unlike high-risk corrective surgery	
Disadvantages	
Safe and satisfactory performance of the procedure requires the use of cardiopulmonary bypass, though a closed procedure should be possible with the use of cutting balloons	
Adequate tailoring of the division and resection can be difficult	
Pulmonary over-flooding is a possibility, in which it should be possible to go on CPB again and close the VSD	

In pediatric cardiac surgery, especially with patients of TOF, understanding the pulmonary arterial anatomy is crucial for making decisions [9]. Brock originally proposed that a rise in blood flow through the repair may stimulate substantial thickening around the pulmonary artery and root. This, subsequently meant that fewer children with TOF will achieve total correction due to significant outflow obstruction. This may increase their likelihood of having right ventricular failure upon eventual closure of the septal lesion or dictate surgically dividing the pulmonary annulus leading to severe pulmonary regurgitation.

## Conclusions

The surgical relief of pulmonary stenosis can be done using direct and indirect procedures. The indirect procedures are associated with dangers; however, the direct Brock's approach seems to be safer by allowing a more normal course of circulation.
